# A nationwide survey of medical resource usage for cancer treatment using Japanese health claims data from 2011 to 2022

**DOI:** 10.1038/s41598-026-55013-x

**Published:** 2026-06-15

**Authors:** Keita Fukuyama, Yukiko Mori, Hiroaki Ueshima, Shigemi Matsumoto, Manabu Muto, Tomohiro Kuroda

**Affiliations:** 1 Division of Medical Information Technology and Administration Planning, Kyoto University Hospital, Kyoto, Japan; 2 Center for Innovative Research and Education in Data Science, Institute for Liberal Arts and Sciences, Kyoto University, Kyoto, Japan; 3 Department of Real World Data Research and Development, Graduate School of Medicine, Kyoto University, Kyoto, Japan; 4 Department of Medical Oncology, Graduate School of Medicine, Kyoto University, Kyoto, Japan

**Keywords:** Cancer therapy, Health resource utilization, Real-world data, Antineoplastic agents, The national database of health insurance claims and specific health checkups of Japan, Cancer, Health care, Oncology

## Abstract

Multidisciplinary cancer treatments have advanced significantly, especially with molecular targeted therapies improving prognosis. Although these are expensive, their cost impact remains unverified. This study aimed to investigate the overall utilization of healthcare resources and identify the cost drivers of cancer treatment in Japan. In this retrospective cohort study, patients diagnosed with malignancies between April 2011 and March 2022 were classified by cancer type, and monthly insurance claims and applicable medications were analyzed. Breast cancer was the most common, followed by stomach and colon cancers. Cost analysis by cancer type revealed that lung cancer was the most expensive overall, whereas multiple myeloma was the most expensive per person-month. When antineoplastic drug costs were aggregated by mechanism, a rapid increase was observed in the immune checkpoint inhibitor category. Since the advent of immune checkpoint inhibitors, the number of insurance claims for antineoplastic drugs in Japan has increased. Japan’s public healthcare system has seen an increase in the cost of cancer treatment at a rate exceeding national economic growth. These findings highlight the need for policies that address the cost-effectiveness and sustainability of cancer care to ensure continued access to high-quality treatment within publicly funded systems in Japan and globally.

**Supplementary Information:**

The online version contains supplementary material available at https://doi.org/10.1038/s41598-026-55013-x.

## Introduction

Cancer is the leading cause of non-communicable disease deaths worldwide, with 20 million new cases and 9.7 million deaths estimated in 2022.^1^ Globally, approximately one in five people will develop cancer in their lifetime, and approximately one in nine men and one in 12 women will die of cancer.^2^ The number of patients with cancer is increasing owing to the aging global population.^2^ Effective cancer treatment requires a multifaceted approach that utilizes various medical resources, including diagnostic tests, surgery, chemotherapy, and radiation therapy. Recently, developments in molecular targeted therapy and immunotherapy have led to favorable treatment outcomes for several cancers, such as tyrosine kinase inhibitors or immune checkpoint inhibitors for non-small cell lung cancer,^3^ hepatocellular carcinoma,^4^ and renal cell carcinoma.^5^ However, the drugs used in these treatments are generally expensive, and there are concerns that cancer treatment will place a strain on health insurance finances.^6^ In a cross-cancer study, Chen et al.^7^ reported that in several countries, the highest estimated costs of cancer were for tracheal, bronchial, and lung cancers, followed by colorectal, breast, and liver cancers. However, these results were compiled using treatment costs for each cancer type in the United States of America, limiting their generalizability to other countries. Shen et al. conducted a cost analysis of hospitalized patients with cancer in Anhui Province, China, but they were unable to evaluate cases in which expensive chemotherapy drugs were used in outpatients.^8^

Japan is one of the world’s leading nations in terms of declining birth rates and an increasingly aging population, with one in two individuals developing cancer and one in four men and one in six women dying from cancer.^9^ Almost all cancer treatments are covered by public insurance; therefore, assessing the economic burden of cancer treatment in Japan can provide valuable insights for policy planning in other countries with aging populations and publicly funded healthcare systems. However, there are several technical challenges in examining the economic burden of cancer and its treatment nationwide. First, medical treatment data from a single hospital would only consider the medical treatment portion of patients’ care, and it is not possible to aggregate the burden of an entire nation. Second, the scale of data on cancer medical treatment costs across the nation is large, and it is technically difficult to aggregate the medical procedures performed on each patient each day. Finally, the prices of drugs and procedures change over time, and it is impossible to aggregate the economic burden without considering these changes.

The National Database of Health Insurance Claims and Specific Health Checkups of Japan (NDB) has been in use since April 2009. The NDB has recorded more than 95% of electronic claims, including medication, medical procedures, examinations, and claims from hospitals, since April 2011.^10^ This database is widely used to verify the status of medical expenses in Japan.^11–13^ Therefore, using data from the NDB, we aimed to create a master database over time to investigate the overall utilization of healthcare resources for cancer treatment in Japan and to identify the cost drivers of cancer treatment. To achieve this, we analyzed data on medical expenditures and medication prescriptions from patients with cancer. This information will be valuable to policymakers, healthcare providers, and researchers in Japan and other countries that subsidize healthcare, aiding in developing strategies to optimize resource allocation and improve the efficiency of cancer treatment while maintaining high-quality care.

## Methods

### Ethics statement

This study was approved by the Ethics Committee of the Kyoto University Graduate School of Medicine (http://www.ec.med.kyoto-u.ac.jp/; approval number R3689). The requirement for informed consent was waived by the ethics committee, as this study utilized anonymized administrative claims data, in accordance with Article 16, Paragraph 2 of the Act on Securing Medical Care for the Elderly in Japan. All procedures performed in studies involving human participants were in accordance with the Declaration of Helsinki 1964 and any subsequent amendments or equivalent ethical standards.

### Study design and data sources

This was a retrospective observational study with anonymized information obtained from the NDB. Data were extracted and provided for all patient IDs associated with benign or malignant tumors from April 2011 to March 2022. We extracted and analyzed IDs linked with only malignant tumors from the provided dataset. We used ID1n, a hash value generated from the insurer’s ID, beneficiary’s ID, birth date, and sex, as patient identifiers. The NDB does not allow for IDs, such as an insured person’s ID, to be analyzed as a person identifier directly. Sex was used as the sex code in the medical insurance registry.

### Study setting and population

In this study, patients with cancer codes were regarded as patients with cancer. Japanese insurance systems require the recording of corresponding injury/disease codes when performing medical treatment. These injury and disease codes are mapped to the International Classification of Diseases, Tenth Revision, and linked to the disease corresponding to the Cancer Incidence in Five Continents (CI5) repository. The CI5 database provides detailed information on the incidence of cancer recorded by population-based cancer registries (https://ci5.iarc.fr/).^14^ The number of patients with CI5 terms was compared with the number of patients extracted from the NDB using Spearman’s correlation index.

### Drug categories

To create a master database that captured changes over time, we created a secondary use of master data published on the Social Insurance Medical Fee Payment Fund website (https://www.ssk.or.jp/seikyushiharai/tensuhyo/index.html).^15^ The master file posted on the Payment Fund website was downloaded recursively and used as the data source. As prices in Japan are reflected from the second day of the month following the month in which the master file was published, prices were assigned by date and not monthly. These data were output in the JavaScript Object Notation format for easy use on various analysis platforms. The code and master code are available on GitHub (https://github.com/fk506cni/ndb_master_creation).

To validate the prices of drugs consumed by drug class, correspondence with the Anatomical Therapeutic Chemical (ATC) classification was obtained from the Kyoto Encyclopedia of Genes and Genomes (KEGG). The KEGG is a database resource for understanding the high-level functions and utilities of biological systems.^16–18^ The correspondence between antineoplastic agents and ATC classification in this study is shown in Supplementary Table S1.

### Time series of medical costs

The total monthly medical billings for patients mapped to each type of cancer, number of patients, and cost per patient per month were verified. The cost of drugs was tabulated by cancer type and age group and presented as the product of the amount used and the drug price on that day. Japan’s final economic growth rate for the corresponding period was calculated from data published by the Japanese Cabinet Office, taking the gross domestic product (GDP) in the first quarter of 2011 as the growth rate for the second quarter of 2022 (https://www.esri.cao.go.jp/en/sna/menu.html).

### Statistical analysis and environment

The dataset provided by the Ministry of Health, Labor, and Welfare (MHLW) was stored in MongoDB (https://www.mongodb.com/), which was configured in a closed network, using Python (https://www.python.org/) for database construction, Julia (https://julialang.org/) for database analysis, and R (https://www.r-project.org/) for visualization. Each process was executed in a corresponding Docker container (Docker Inc., Palo Alto, CA), and the Docker file for analysis is available on GitHub (https://github.com/fk506cni/ndb_pan_cancer/tree/main/dockerfile).

## Results

### Drug price distribution

**Fig. 1:**
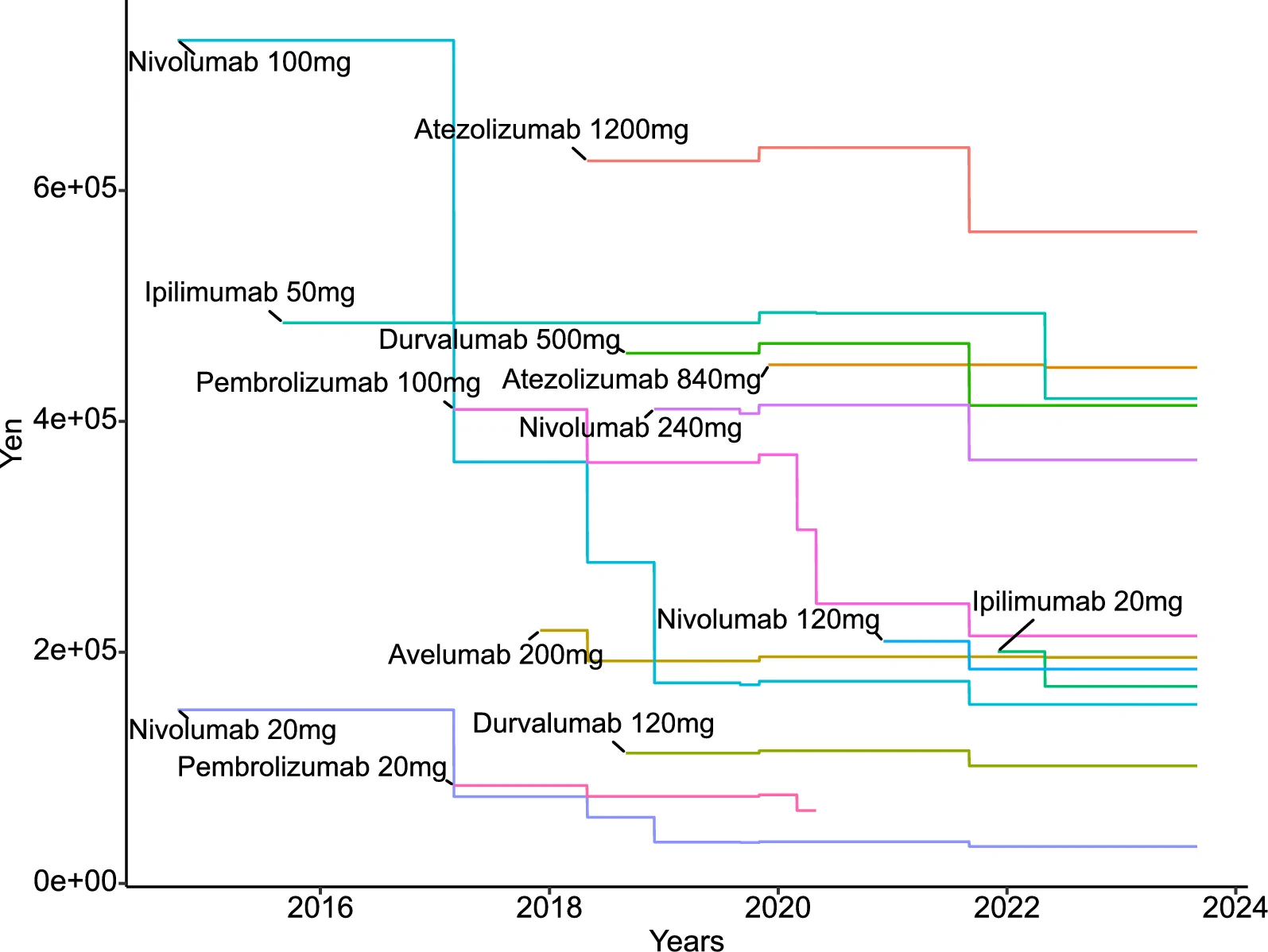
Trends in the price of immune checkpoint inhibitors. Each color indicates the drug price for one vial or ampoule at several time points.

A master reflecting price trends over time was created for 31,587 drugs, 23,182 procedures, and 4,659 specific equipment items. Immune checkpoint inhibitors are one of the most innovative drug groups in cancer treatment in recent years, and their introduction has raised concerns that they could contribute to overall drug cost increases. We selected this group of drugs to illustrate the complexity of price fluctuations due to the multiple drugs on the market and frequent drug price revisions in Japan (Fig. 1). Even among immune checkpoint inhibitors, the decline in price trends was not uniform, contributing to the difficulty of accurately evaluating the burden of drugs on medical economics.

### Patient distribution

**Fig. 2:**
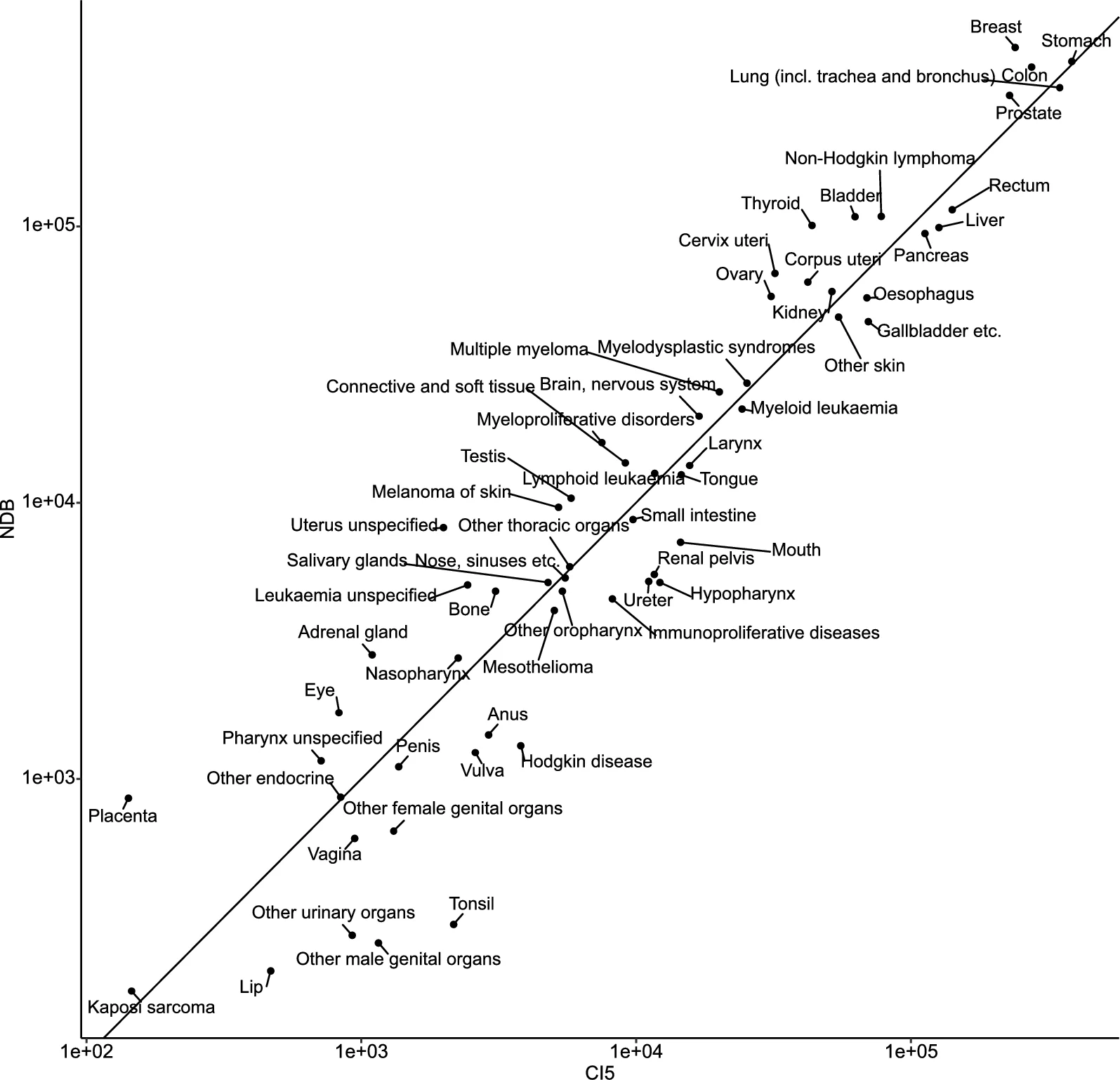
Comparison of NDB and CI5 data from 2013–2015. The number of patients with each cancer type defined by the ICD-10 code and CI5 master data is depicted. The black line indicates CI5 = NDB.

Cancer patient receipt data for 23.2 million individuals and 51.9 million person-months were collected among all the NDB data from 2011 to 2022. The flowchart of patient selection is shown in Supplementary Fig. 1. The distribution of the number of patients per cancer type, including colon, breast, prostate, and lung cancers, in 2013–2015 compared with the Japanese CI5 data for the 2013–2015 period is shown in Fig. 2. The correlation coefficient between the two data sources was 0.932. The distribution of patients with cancer was highly correlated with the CI5 data. The distribution of age, sex, and cancer type for all patients in 2011–2022 is shown in Supplementary Table S2. In total, 23.2 million people were analyzed, including 11.2 million women and 12 million men. The most common age group was 75–79 years (24%), and the most common cancers were breast cancer in women (2.6 million people, 23.3%) and prostate cancer in men (1.81 million people, 15.0%). Regarding the distribution of the number of patients for each cancer type, results equivalent to those of CI5 data were observed, and this is considered to reflect the actual distribution of patients with cancer in Japan.

### Total cost distribution

**Fig. 3:**
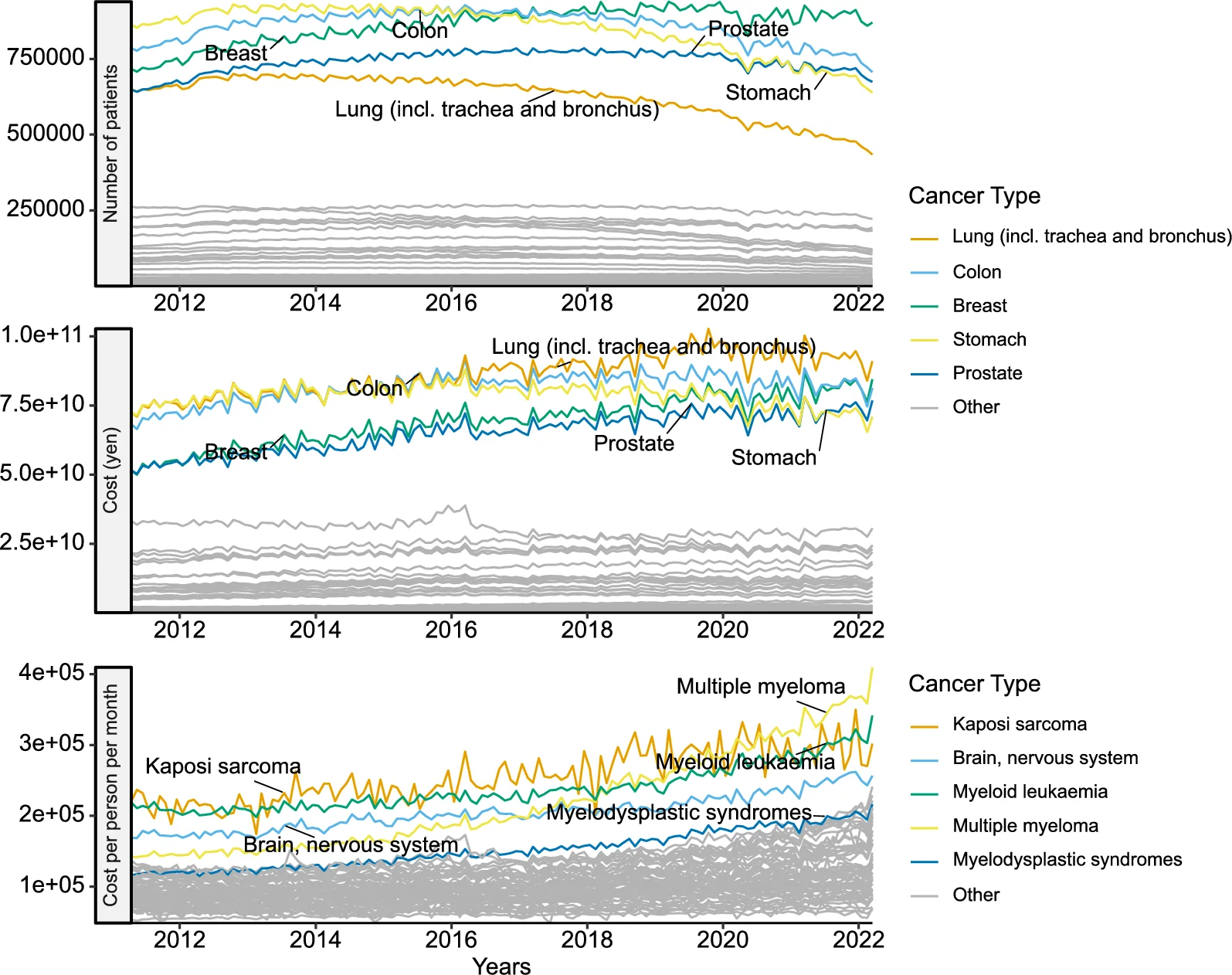
Number of patients (person-months), billing costs, and cost per person-month for patients with a single cancer. Colors indicate the respective cancer types. Cancers outside the top five categories are shown in gray as "other."

The distribution of the costs according to cancer type among patients with a single cancer is shown in Fig. 3. The distribution of usage of each drug by patients across cancer types is presented in Supplementary Table S3. The cancer type with the highest cost was “lung (including trachea and bronchus)” in the final fiscal year (ending in March 2022), despite the number of patients decreasing. In contrast, “multiple myeloma” was the most expensive in terms of the amount billed per person per month (409,000 yen per person-month as of March 2022). The number of patients, claims costs, and cost distribution per person-month for all cancer types, including cases of multiple cancers, are shown in Supplementary Figure S2. Although the number of patients with cancer for whom medical fee claims had been filed had decreased (-680,000) from April 2011 (6.12 million) to March 2022 (5.44 million), the total billing costs had increased by 151 billion yen, from 559 billion yen to 710 billion yen.

**Fig. 4:**
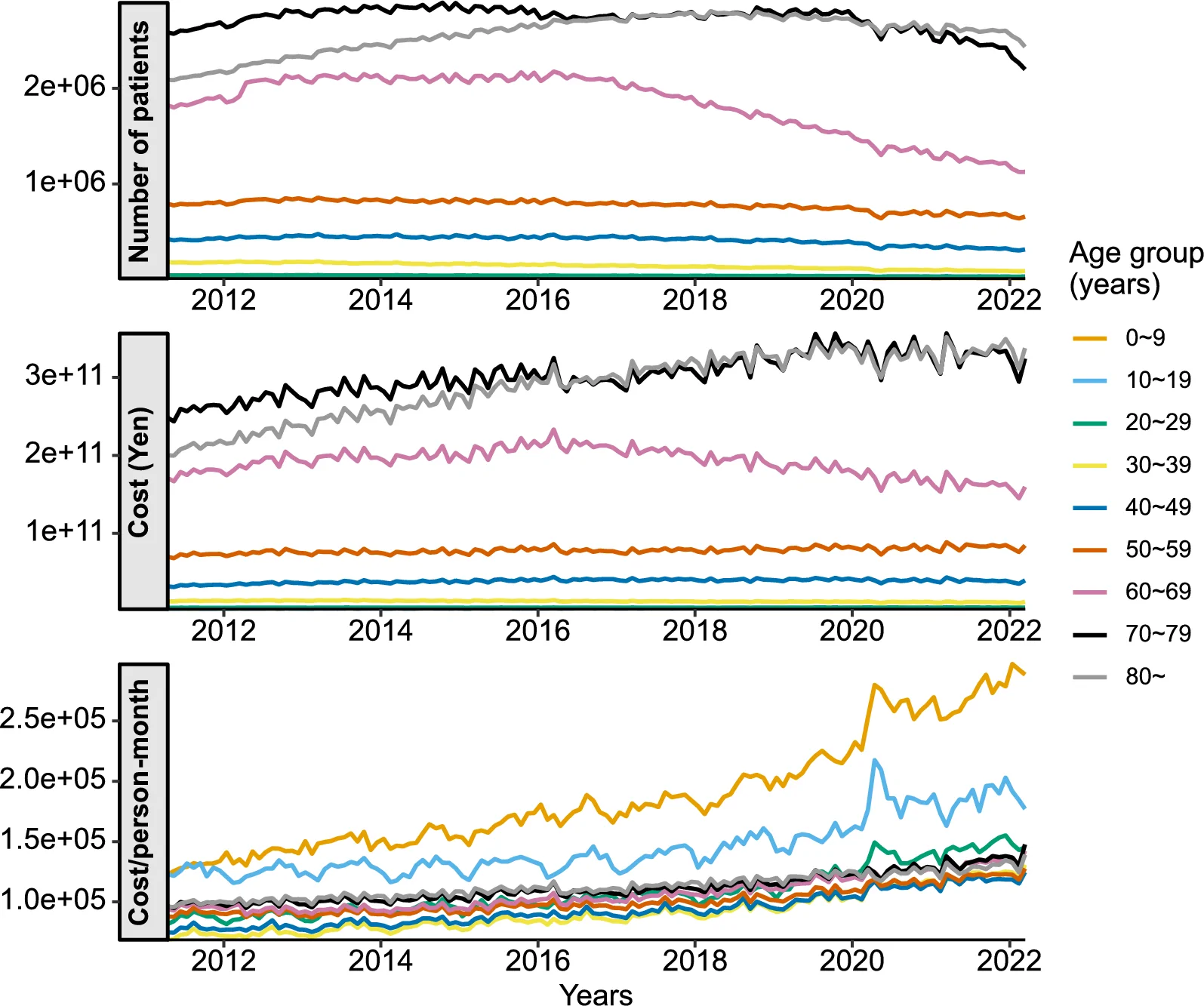
Number of patients with cancer (person-months), claim costs, and cost per person-month for each age group. Colors indicate the respective age groups.

An analysis of the medical claims from patients with cancer by age group showed that the total cost was highest for those aged 70–80 years and 80 years and older, which accounted for most claims (Fig. 4). However, when evaluated on a per-person-month basis, the treatment of children was the most expensive.

### Antineoplastic drug cost distribution

**Fig. 5:**
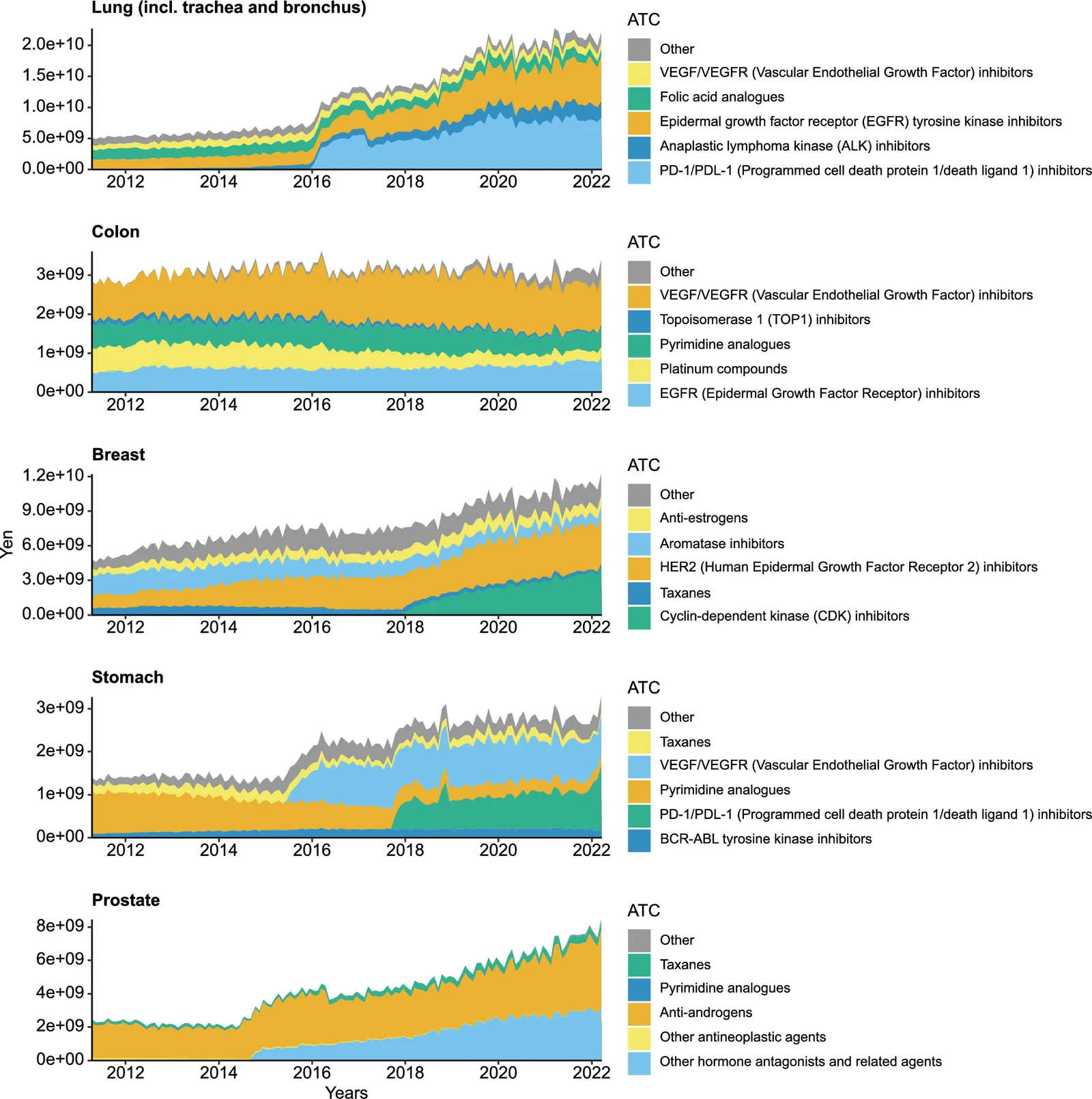
Cost distribution of each cancer drug for the five major cancer types. Area colors indicate the drug category. Drug categories other than the top five categories for each cancer are shown in gray as "others." The color correspondence varies for each cancer type. ATC, Anatomical Therapeutic Chemical classification.

The cost distribution of antineoplastic drugs for the top five cancers with respect to cost claims is shown in Fig. 5. There was a significant rise (16.94 billion yen per month) in the cost of lung cancer treatment from April 2011 (5.06 billion yen) to March 2022 (22 billion yen). For cancers other than colorectal cancer, anticancer drug costs were on the rise. Agents increasing the overall costs were programmed cell death protein 1/death ligand 1 (PD-1/PDL-1) inhibitors for lung cancer, human epidermal growth factor receptor 2 inhibitors and cyclin-dependent kinase inhibitors for breast cancer, and vascular endothelial growth factor (VEGF)/VEGF receptor inhibitors and PD-1/PDL-1 inhibitors for gastric cancer. For prostate cancer, other hormone antagonists and related agents increased overall costs.

**Fig. 6:**
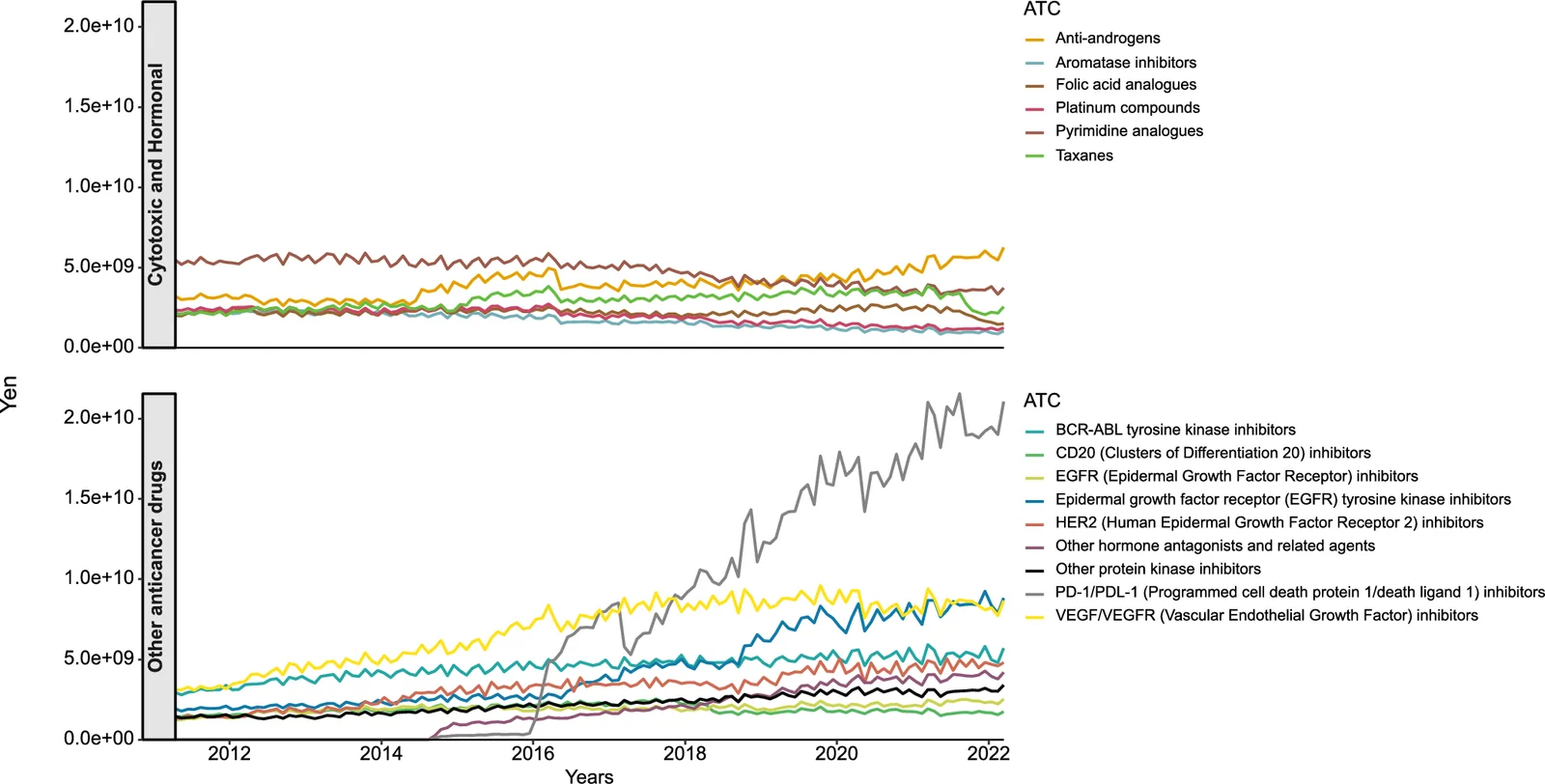
Distribution of costs across antineoplastic drugs. The line colors indicate the drug category. ATC, Anatomical Therapeutic Chemical classification. Drug categories with a total value of less than 200 billion yen are not plotted.

In addition, the distribution of costs across antineoplastic drugs showed a marked increase in PD-1/PDL-1 inhibitor drugs (Fig. 6). The monthly total price trends for all anticancer drugs are shown in Supplementary Figure S3. From April 2011 (34.7 billion yen) to March 2022 (109 billion yen), there was an increase of 73.9 billion yen.

## Discussion

The financial burden of cancer has been described as financial toxicity, focusing on the patient burden.^19^ To protect citizens from financial hardships associated with cancer and its treatment, many countries cover the cost of high-priced drugs through public funding. However, the financial burden of these drugs on public medical insurance for several cancer types has not been verified. This study reports the trends in medical costs associated with cancer treatment over the past 11 years in Japan under the public healthcare insurance system, focusing on antineoplastic drug treatment. Since the advent of immune checkpoint inhibitors and several molecular targeted therapies, the overall cost of antineoplastic drug treatment, particularly within these categories, has dramatically increased. Although the prices of some PD-1/PDL-1 inhibitors have declined, overall, such drugs have generated extremely high costs. Epidermal growth factor receptor tyrosine kinase inhibitors have followed this pattern, and VEGF/VEGF receptor inhibitors remain expensive. Although the cost of certain drugs has declined, the total cost of anticancer drugs and total billing cost increased approximately 3.1-fold (from 34.7 billion to 109 billion yen) and 1.27-fold (from 559 billion yen to 710 billion yen), respectively, during the study period.

**Fig. 7:**
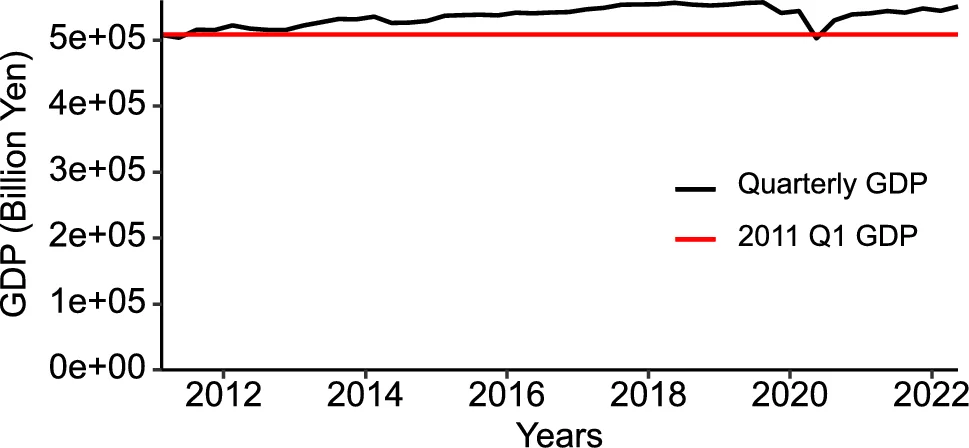
Economic development of Japan from the first quarter of 2011 to the second quarter of 2022. The black line indicates the Quarterly GDP, and the red line indicates the 2011 Q1 GDP baseline.

In Japan, the economic growth development was 8.5% from the first quarter of 2011 (508.2 trillion yen) to the second quarter of 2022 (551.2 trillion yen) (Fig. 7). The rate of increase in the cost of insured cancer treatment and cancer chemotherapy exceeds the rate of economic growth in Japan, and if this trend continues, cancer treatment is likely to have a significant impact on national finances. Although previous studies have examined medical costs, they have not compared the economic changes of the target countries.^7,8^ When evaluating the sustainability of publicly insured medical care, it is essential to consider the economic situation of the country that ultimately bears the financial burden.

Importantly, our results do not suggest refraining from using these drugs solely based on cost. The efficacy of these agents has been demonstrated in clinical trials, and they are used as effective agents. However, much of the financial burden is covered by public funds, and the current rate of increase in the cost of cancer treatment exceeds the rate of economic development of Japan. Because many of the expensive new drugs are used for advanced or recurrent cancer, in order to reduce the impact of these drugs on the medical economy, it is important to prevent the onset of cancer or to detect it early and apply more radical and inexpensive treatment (e.g., endoscopic resection, surgery). Therefore, an evaluation of the cost-effectiveness of other interventions, such as early detection through cancer screenings or prevention through vaccinations, is advisable.^20^

Japan began implementing a cost-effectiveness evaluation system in April 2019. The cost-effectiveness evaluation of new drugs utilizes the Incremental Cost-Effectiveness Ratio (ICER), which is calculated by dividing the “additional cost” incurred when using a new drug or medical device by the “additional benefit” gained. The ICER threshold in Japan is generally set at 5 million yen/quality-adjusted life year.^21^ Meanwhile, real GDP per capita from fiscal year 2011 to fiscal year 2022 ranged from 4.02 million yen to 4.42 million yen,^22^ corresponding to an ICER threshold of 1.13 to 1.24 of GDP per capita.

As shown in Fig. 5, the introduction of new drugs often affects chemotherapy costs by incurring additional costs rather than replacing the costs of other drugs. Furthermore, as shown in Supplementary Figure S3, whole antineoplastic drug costs have continued to increase since April 2019. Our results show that an evaluation based on ICER using a threshold approximately 1.2 times GDP per capita does not adequately control for the increase in the cost of cancer chemotherapy.

In Japan, as of September 2023, only 28 drugs had completed cost-effectiveness evaluation, while 17 are still undergoing evaluation.^23^ In addition, we found no reports of cost-effectiveness analyses for insurance coverage that considers the nation’s economic growth and sustainability of insured medical care beyond the cost-effectiveness of individual patients. A system is currently being developed for the large-scale collection and utilization of diverse data, including clinical outcomes, from digitized medical settings, forming the foundation for next-generation medical research, medical systems, and medical administration driven by digital data.^24^ This framework is expected to facilitate the selection of cost-effective treatments while considering the sustainability of insured medical care.

## Limitations

This study has some limitations. First, cancer classification was based on the disease name listed on medical claims. While the NDB cancer classification showed a high correlation compared with CI5, some cancers showed substantial deviation, which may be explained by several factors. Disease naming in health insurance-covered medical care is used to justify medical treatment and does not require the same rigorous evaluation as cancer registries. For this reason, categories such as C57: Other female genital organs and C63: Other male genital organs may overlap with similar categories such as C56: Ovary and C62: Testis. Furthermore, for breast cancer patients, whose treatment periods tend to be long, the number of patients is likely to be overestimated owing to changes in IDs. Therefore, a certain degree of systematic error in the number of patients based on strict cancer classification and their distribution on medical claims should be considered.

Second, for patients with multiple cancers, for example, a patient with both breast and lung cancers, it is not possible to accurately estimate the receipt information in which cancer is the main source of healthcare expenditures. Therefore, patients with multiple cancers are categorized separately from patients with a single cancer. There is a concern that cancers with a large number of patients, such as breast and lung cancers, may be more likely to be included in the multiple cancers category. Therefore, the medical cost of these cancers may be underestimated.

Third, this study only evaluated chemotherapy, and it did not assess technological innovations and the associated changes in costs for surgical treatment and radiotherapy, such as robotic surgery against open radical surgery or intensity-modulated radiation therapy against conventional radiotherapy. Although masters have been created for reimbursement associated with these medical procedures over time, their costs have been excluded from the present validation, as it is difficult to aggregate their financial burden from reimbursement for procedures alone. Lastly, this study examined only Japan, and the impact of cancer treatment costs on national finances in each country should be examined based on the system of each country. However, the prices of anticancer drugs are similarly high in other countries,^6^ making the findings of this study very relevant for policymakers, healthcare providers, and researchers not only in Japan but also in other countries where healthcare is subsidized by public funds.

## Conclusions

The medical resources utilized by patients with cancer for health insurance treatment are increasing at a rate that exceeds Japan’s economic growth. Further research for indicators that will enable insurance reimbursement decisions to be made in line with the scale of the economy is required to keep public expenditures within a sustainable range.

## Supplementary Information


Supplementary Information 1.
Supplementary Information 2.
Supplementary Information 3.
Supplementary Information 4.


## Data Availability

The raw data that support the findings of this study are available from the Ministry of Health, Labour and Welfare (MHLW) of Japan, but restrictions apply to their availability. Under Japanese law (Article 16, Paragraph 2 of the Act on Securing Medical Care for the Elderly), the authors are strictly prohibited from distributing the raw national health claims data (NDB) to third parties to protect patient privacy. Researchers wishing to access the raw NDB data must apply directly to the MHLW (https://www.mhlw.go.jp/stf/seisakunitsuite/bunya/kenkou_iryou/iryouhoken/reseputo/index.html). Aggregated summary data that support the findings of this study and have been approved by the MHLW are available within the article and its Supplementary Information files. Requests for intermediate processed data may be directed to the corresponding author (Keita Fukuyama); however, sharing is strictly contingent upon formal approval by the MHLW on a case-by-case basis. The specific conditions for obtaining such permissions are governed by the MHLW’s rigorous national standards, which include: (1) Eligible Institutions: Access is limited to public agencies, universities, and approved research institutions. (2) Public Interest & Data Minimization: The research must demonstrate a clear public benefit, and data extraction is limited to the minimum necessary variables. (3) IRB Approval: Prior approval from an Institutional Review Board (IRB) or equivalent ethics committee is mandatory. (4) Strict Data Security: Researchers must implement rigorous physical and IT security measures (e.g., isolated terminals and security audits). (5) Strict Publication Rules: All publications must adhere to strict reporting criteria to prevent patient re-identification. Detailed official requirements and manuals can be found at: Official Manual for Data Utilization: https://www.mhlw.go.jp/content/12400000/001679194.pdf Official Guidance for Data Utilization: https://www.mhlw.go.jp/content/12400000/001588802.pdf To facilitate reproducibility, the entire analysis pipeline—including codes, queries, and processing notebooks—is publicly available on GitHub (https://github.com/fk506cni/ndb_pan_cancer/).

## Abbreviations

ATC: Anatomical therapeutic chemical
CI5: Cancer incidence in five continents
GDP: Gross domestic product
ICD-10: International classification of diseases, Tenth revision
KEGG: Kyoto encyclopedia of genes and genomes
MHLW: Ministry of health, labor, and welfare
NDB: National database of health insurance claims and specific health checkups of Japan
PD-1: Programmed death-1
PDL-1: Programmed death ligand-1
VEGF: Vascular endothelial growth factor
