# Supplementary material for: A nationwide survey of medical resource usage for cancer treatment using Japanese health claims data from 2011 to 2022
Source: Sci Rep. 2026 Jun 15;16:27362. doi: 10.1038/s41598-026-55013-x (PMC13530265; doi:10.1038/s41598-026-55013-x)
Supplement: Supplementary file 1 [file 41598_2026_55013_MOESM1_ESM.pdf]

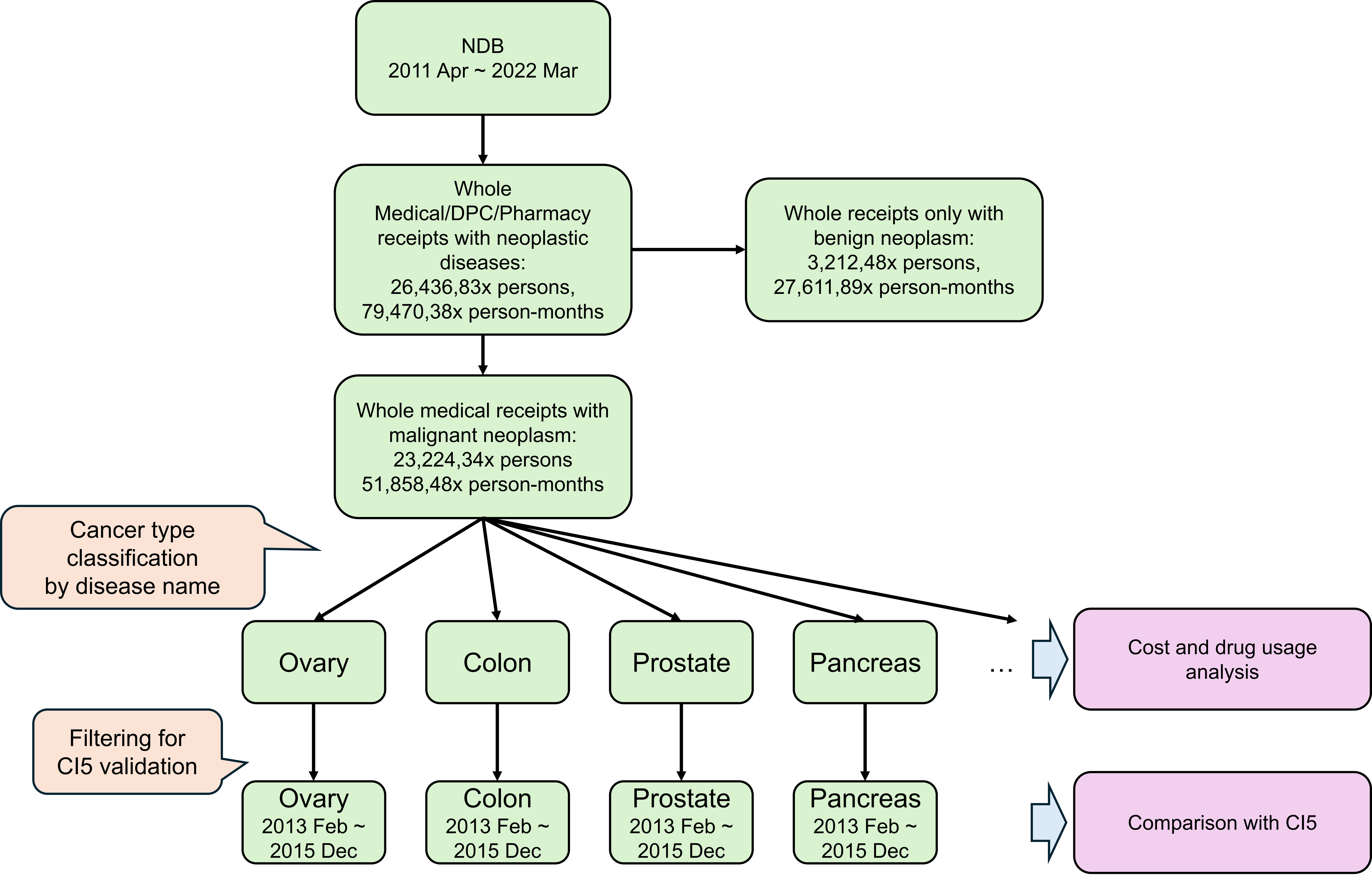
NDB
2011 Apr ~ 2022 Mar
Whole Medical/DPC/Pharmacy receipts with neoplastic diseases:
26,436,83x persons,
79,470,38x person-months
Whole receipts only with benign neoplasm:
3,212,48x persons,
27,611,89x person-months
Whole medical receipts with malignant neoplasm:
23,224,34x persons
51,858,48x person-months
Cancer type classification by disease name
Ovary
Colon
Prostate
Pancreas
…
Cost and drug usage analysis
Filtering for CI5 validation
Ovary 2013 Feb ~ 2015 Dec
Colon 2013 Feb ~ 2015 Dec
Prostate 2013 Feb ~ 2015 Dec
Pancreas 2013 Feb ~ 2015 Dec
Comparison with CI5
