# Supplementary material for: A nationwide survey of medical resource usage for cancer treatment using Japanese health claims data from 2011 to 2022
Source: Sci Rep. 2026 Jun 15;16:27362. doi: 10.1038/s41598-026-55013-x (PMC13530265; doi:10.1038/s41598-026-55013-x)
Supplement: Supplementary file 2 [file 41598_2026_55013_MOESM2_ESM.pdf]

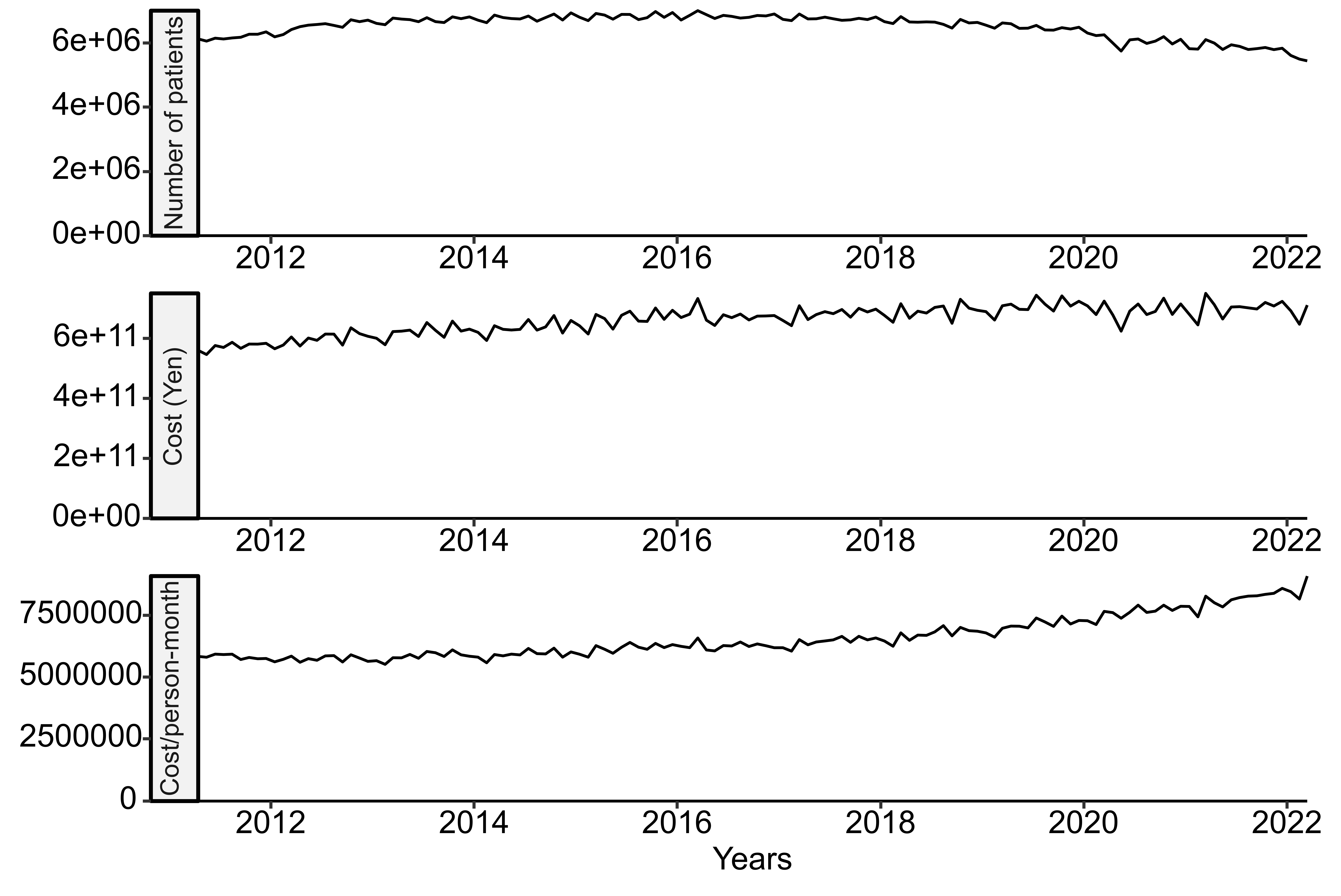
Number of patients
6e+06
4e+06
2e+06
0e+00
Cost (Yen)
6e+11
4e+11
2e+11
0e+00
Cost/person-month
7500000
5000000
2500000
0
2012
2014
2016
2018
2020
2022
Years
