# Supplementary material for: A nationwide survey of medical resource usage for cancer treatment using Japanese health claims data from 2011 to 2022
Source: Sci Rep. 2026 Jun 15;16:27362. doi: 10.1038/s41598-026-55013-x (PMC13530265; doi:10.1038/s41598-026-55013-x)
Supplement: Supplementary file 3 [file 41598_2026_55013_MOESM3_ESM.pdf]

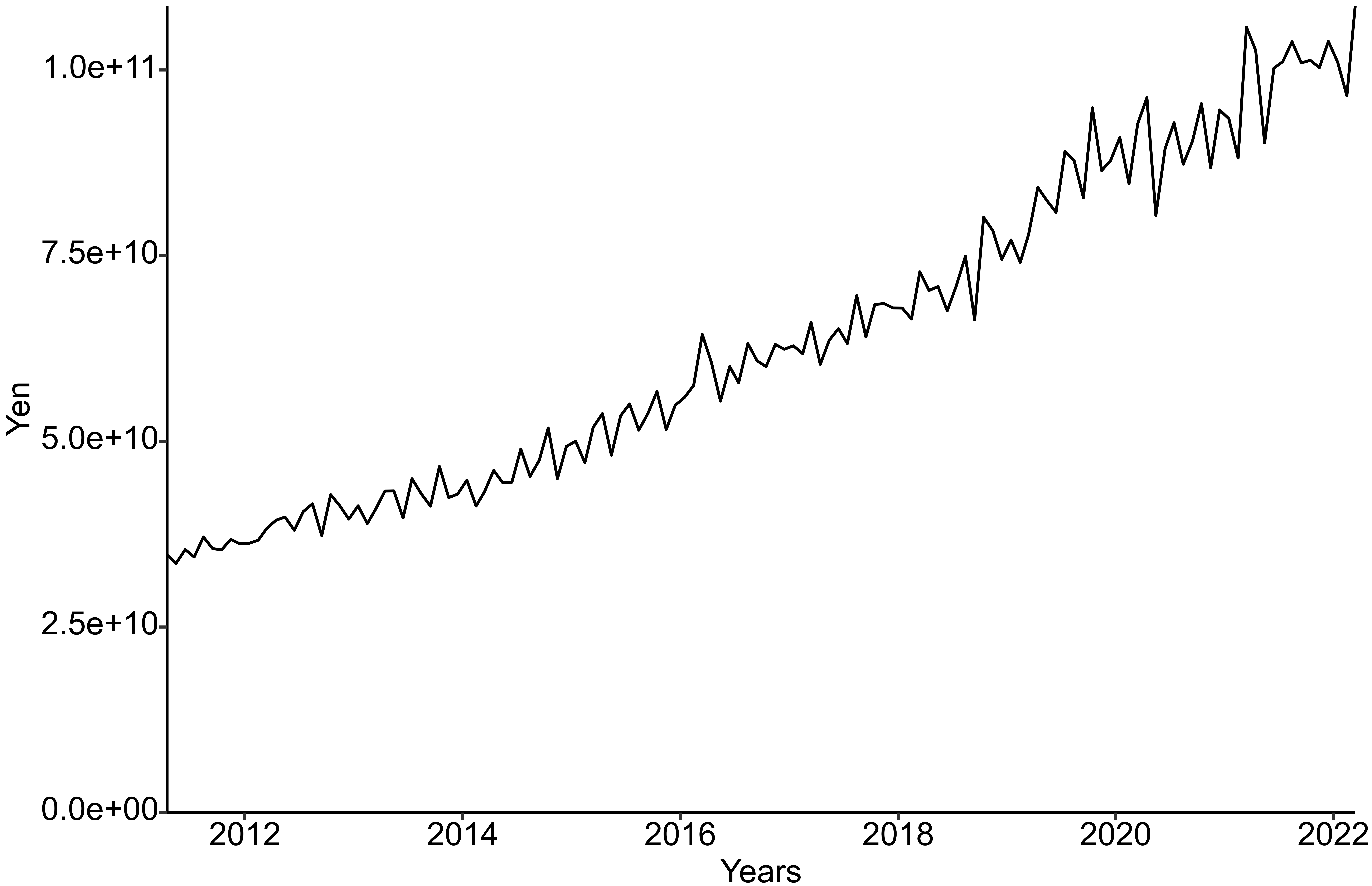
1.0e+11
7.5e+10
5.0e+10
2.5e+10
0.0e+00
Yen
2012
2014
2016
2018
2020
2022
Years
